# Accuracy of spiked cell counting methods for designing a pre-clinical tumorigenicity study model

**DOI:** 10.1016/j.heliyon.2020.e04423

**Published:** 2020-07-13

**Authors:** Hiroaki Osada, Masahide Kawatou, Masafumi Takeda, Jun-ichiro Jo, Takashi Murakami, Yasuhiko Tabata, Kenji Minatoya, Jun K. Yamashita, Hidetoshi Masumoto

**Affiliations:** a Department of Cardiovascular Surgery, Graduate School of Medicine, Kyoto University, Kyoto, Japan; b Department of Cell Growth and Differentiation, Center for iPS Cell Research and Application, Kyoto University, Kyoto, Japan; c Institute for Advancement of Clinical and Translational Science, Kyoto University Hospital, Kyoto, Japan; d Laboratory of Biomaterials, Department of Regeneration Science and Engineering, Institute for Frontier Life and Medical Sciences, Kyoto University, Kyoto, Japan; e Department of Microbiology, Saitama Medical University, Faculty of Medicine, Saitama, Japan; f Clinical Translational Research Program, RIKEN Center for Biosystems Dynamics Research, Kobe, Japan

**Keywords:** Biochemistry, Cell biology, Tissue engineering, Cell culture, Stem cells research, Biomedical engineering, Regenerative medicine, Stem cell therapy, Tumorigenicity, Pre-clinical safety tests, Methodology

## Abstract

**Background:**

Evaluations for the tumorigenicity of transplantation of stem cell products is mandatory for clinical application. It is of importance to establish a system to accurately quantify contaminated tumorigenic cells regardless of the format of stem cell product. In the present report, we aimed to examine the accuracy of the quantification of tumorigenic cell numbers with commonly used 2 methods, quantitative polymerase chain reaction (qPCR) and flow cytometry (FCM) using experimental models of stem cell products spiked with tumorigenic cells.

**Methods:**

Human mesenchymal stem cells (hMSCs) and melanoma Mewo-Luc cells constitutively expressing luciferase were used. We stained Mewo-Luc cells with a cell linker then spiked onto hMSC suspensions and hMSC sheets. We validated the accuracy of 10-fold serial dilution technique for Mewo-Luc cell suspension using a Coulter counter. The samples spiked with Mewo-Luc cells were subjected to qPCR and FCM analyses, respectively for the quantification of Mewo-Luc cells.

**Results:**

Ten-fold serial dilutions of Mewo-Luc cells were performed accurately with small deviation. In samples spiked with or less than 100 cells in hMSC suspensions, and samples spiked with or less than 1,000 cells in hMSC sheets showed significantly higher cell numbers in calculations by FCM, respectively (suspensions; qPCR vs FCM: 100 cells: 59 ± 25 vs 232 ± 35 cells, p = 0.022/10 cells: 21 ± 7 vs 114 ± 27 cells, p = 0.030, sheets; qPCR vs FCM: 1,000 cells: 1723 ± 258 vs 5810 ± 878 cells, p = 0.012/100 cells: 110 ± 18 vs 973 ± 232 cells, p = 0.012/10 cells: 20 ± 6 vs 141 ± 36 cells, p = 0.030).

**Conclusion:**

Differences in accuracy between quantification methods should be considered in designing a tumorigenicity study model.

## 1 Introduction

Stem cell products manufactured from various stem cell populations (e.g. bone marrow-derived hematopoietic stem or stromal cells, skeletal myoblasts, pluripotent stem cells) are being increasingly applied for clinical use worldwide [1, 2, 3, 4, 5]. However, stem cell products are associated with risks for tumor formation after transplantation which are potentially attributed by disorganized proliferation of mitogenic cells or malignant transformation of transplanted cells [6]. To standardize stem cell transplantation therapy, it is crucial to establish an appropriate evaluation method for so called “tumorigenicity” of stem cell products.

Tumorigenicity is defined as a capacity of cells inoculated into an animal model to generate a tumor at the site of inoculation by local proliferation and/or the proliferation at remote sites by metastasis. To test the tumorigenicity, Technical Report Series 878 of World Health Organization entitled “Recommendation for the evaluation of animal cell cultures as substrates for the manufacture of cell banks” recommends subcutaneous transplantation of 10^7^ of subject cells into 10 immunodeficient nude mice and a monitoring of tumor formation for more than 16 weeks [7, 8]. Transplantation of the same number of well-established tumorigenic cells such as HeLa cells in parallel is recommended as a positive control.

Several studies have proposed methods to evaluate tumorigenicity of stem cell products [9, 10, 11, 12]. One of the studies [11] aimed to identify a 50 % tumor-producing dose (TPD_50_), a dose that generates tumors in 50 % of transplanted mice, which contributes to assess the tumorigenicity of the cell product with high sensitivity. The study examined the percentage of tumor formation according to logarithmically allocated HeLa positive control cell numbers by subcutaneous transplantations onto immunodeficient mice, then TPD_50_ was calculated as a cell number which can generate tumors in 50 % of mice. Not only in the abovementioned study but also in other studies, it is indispensable to quantify tumorigenic cells (which are exogenously spiked in experimental models) contaminated in the products for precise evaluations of the tumorigenicity.

To prepare certain number of positive control cells to spike, serial dilution is commonly used. Cell density of a diluted solution is based on the theory of Poisson distribution [13, 14]. Serial dilution is an essential method to especially prepare small number of cells which cannot be counted by usual cell counting methods. Although feasible serial dilution systems have been reported so far [15, 16], accuracy of the dilutions have not been fully examined. Furthermore, no study has validated the accuracy of serially diluted spiked cell numbers to conduct tumorigenicity studies. Considering various formats of stem cell products such as cell sheets [3] which require incorporation processes of positive control cells during the formation of cell products, it is of importance to establish a system to accurately quantify incorporated positive cells regardless of the format of stem cell product.

In the present study, we aimed to examine the accuracy of the quantification of spiked cell number with commonly used 2 methods [quantitative polymerase chain reaction (qPCR) and flow cytometry (FCM)] in 2 formats of stem cell products [human mesenchymal stem cell (hMSC)-derived cell suspensions and cell sheets] spiked with genetically and fluorescently labelled positive control cells recapitulating malignant transformation [a malignant melanoma cell line constitutively expressing luciferase (Mewo-Luc) labeled with a fluorescent cell linker], respectively.

## 2 Materials and methods

### 2.1 Human mesenchymal stem cells (hMSCs)

hMSCs were purchased from Lonza (Basel, Switzerland) and cultured in MF-medium (TOYOBO, Tokyo, Japan). For the maintenance of hMSCs, the culture media were replenished every 2 days. hMSCs were detached and dissociated into single cell suspension by 4–5 min incubation with trypsin solution [Trypsin/ethylenediaminetetraacetic acid (EDTA) for Mesenchymal Stem Cells, Lonza]. Live cell numbers were manually counted using hemocytometer.

### 2.2 Melanoma cells; Mewo-Luc

Melanoma cells constitutively expressing luciferase (Mewo-Luc; established by one of the authors, T.M.) were purchased from JCRB cell bank (Osaka, Japan) and cultured in alpha minimum essential medium (αMEM; GIBCO, Grand Island, NY, USA) supplemented with 10 % fetal bovine serum (FBS), 5.5 mmol/L of 2-mercaptoethanol, 50,000 U/L of penicillin and 50 mg/L of streptomycin on a non-coated culture dish. For the maintenance of Mewo-Luc cells, the culture media were replenished every 2 days. After 5–7 days of culture, Mewo-Luc cells were detached and dissociated into single cell suspension by 3 min incubation with 0.25 % trypsin solution with EDTA (Life Technologies, Carlsbad, CA, USA). Live cell numbers were manually counted using hemocytometer. We stained Mewo-Luc cells with a fluorescence marker; PKH26 Red Fluorescent Cell Linker Kit (Sigma-Aldrich, St. Louis, MO, USA) following the manufacturer's instruction before cell spiking.

### 2.3 Validation of serial dilution technique

We counted cell number of 10-fold serially diluted single Mewo-Luc cell suspension samples using a Coulter Counter (Multisizer 4e, Beckman Coulter, Inc., Brea, CA, USA) to validate the spiking cell number. Dissociated Mewo-Luc cell suspensions were prepared as 2,000,000 cells/mL counted with hemocytometer. We first analyzed average diameter of Mewo-Luc cell with Vi-CELL XR (Beckman Coulter, Inc.), then prepared 20 mL of specimen (2,000,000 cells/10mL) by mixing 2 mL of the cell suspension (2,000,000 cells/mL) with 18 mL of Coulter Isotone II diluent (Beckman Coulter, Inc.). Serial 10-fold dilutions are conducted with Coulter Isotone II diluent to prepare specimens of 200,000 to 20 cells/10mL (10–100,000-fold dilution). The original and diluted specimens were subjected to cell counting using Multisizer 4e based on the average cell diameter (aperture diameter 100 μm; analysis particle size set as 8.0–60.0 μm). We waited several minutes before each analysis to completely remove micro babbles in the sample. Filtered Coulter Isotone II diluent was used to remove small particle as much as possible. The experiments were repeated 4 times. We calculated serially diluted cell number as per 10mL. Cell numbers were provided as ×0.5 because we spiked half of the diluted solutions in the following experiments.

### 2.4 Preparation of hMSC suspensions and Mewo-Luc cell spiking (Figure 1A)

hMSC suspensions (500,000 cells) were prepared in microtubes. PKH-labeled Mewo-Luc cell suspension was serially diluted from 2,000,000 cells to 2 cells (10-fold serial dilution). Half of the diluted solutions (containing 100,000 cells, 10,000 cells, 1,000 cells, 100 cells, 10 cells or 1 cell, respectively) were spiked to each hMSC suspension tube. The experiments were repeated 5 times.

**Figure 1:**
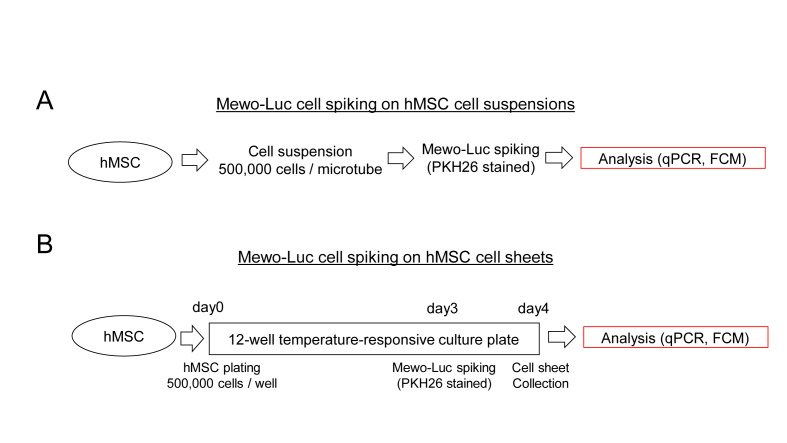
Flow charts of Mewo-Luc cell spiking on stem cell products. (A) Mewo-Luc cell spiking on hMSC cell suspensions. (B) Mewo-Luc cell spiking on hMSC cell sheets. hMSC, human mesenchymal stem cell; qPCR, quantitative polymerase chain reaction; FCM, flow cytometry.

### 2.5 Preparation of hMSC sheets and Mewo-Luc cell spiking (Figure 1B)

We seeded 500,000 hMSCs on the FBS-coated 12-well temperature-responsive culture plates (UpCell, CellSeed, Tokyo, Japan) [17, 18, 19] in αMEM (GIBCO) supplemented with 10 % FBS, 5.5 mmol/L of 2-mercaptoethanol, 50,000 U/L of penicillin and 50 mg/L of streptomycin and incubated at 37 °C. Three days later, PKH-labeled Melanoma Mewo-Luc cells with serial dilution as the same manner as for hMSC suspensions were spiked onto each well. One day later, the sheets were detached at room temperature and dissociated in 0.25 % trypsin solution with EDTA (Life Technologies). Live cell numbers were manually counted using hemocytometer. The experiments were repeated 5 times.

### 2.6 Quantitative polymerase chain reaction (qPCR)

hMSC suspensions and sheets spiked with Mewo-Luc, and cell suspension of pure Mewo-Luc were subjected to qPCR. Total RNA was extracted with RNeasy® Mini Kit (Qiagen, Hilden, Germany) following the manufacturer's instruction. RNA concentration was determined using a NANO DROP 2000C spectrophotometer (Thermo Fisher Scientific, Waltham, MA, USA). Reverse transcription reactions were performed using standard procedures to synthesize first-strand cDNA with SuperScript®III First-Strand Synthesis SuperMix for qRT-PCR (Thermo Fisher Scientific). qPCR was performed using the StepOnePlus Real-Time PCR System (Thermo Fisher Scientific). The expression levels of luciferase were normalized to those of ribosomal 18s RNA. The sequences of gene-specific luciferase and ribosomal 18s primers used in qPCR amplification are as follows (5’ – 3′): luciferase: Forward: TATCCGCTGGAAGATGGAAC, Reverse: CGAAGTACTCAGCGTAAGTG/ribosomal 18s: Forward: CCTTTGCCATCACTGCCATT, Reverse: TGATCACAGGTTCCACCTCA. The percentages of spiked Mewo-Luc cells included in each sample were determined by relative expression levels of luciferase in samples and those in pure Mewo-Luc cells; the expression level of luciferase in pure Mewo-Luc cells was set as 100 %. Mewo-Luc cell count included in a hMSC suspension or sheet based on qPCR was calculated by total live cell number and the percentage of spiked Mewo-Luc quantified by qPCR.

### 2.7 Flow cytometry (FCM)

Mewo-Luc cell suspensions just after PKH-labeling, and hMSC suspensions and sheets spiked with Mewo-Luc were subjected to FCM. FACS Aria II (BD Biosciences, Franklin Lakes, NJ, USA) and FACS Diva software ver. 8.0 (BD Biosciences) were used for analyses. Green phycoerythrin (PE) detector was used for the detection of PKH fluorescence. Mewo-Luc cell count included in a hMSC suspension or sheet based on FCM was calculated by total live cell number and live PKH-positive cell ratio measured by FCM.

### 2.8 Statistical analysis

All data analyses were performed using JMP pro version 14 (SAS Institute, Cary, NC, USA). Statistical analysis of the data was performed with Steel-Dwass multiple-comparison test. P < 0.05 was considered significant. Values are reported as means ± S.E.M.

## 3 Results

### 3.1 Efficiency of fluorescent labeling of positive cells

**Figure 2:**
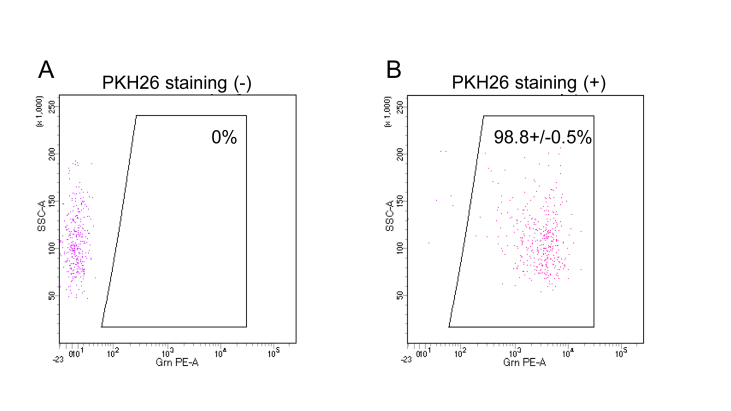
Flow cytometry result of Mewo-Luc cells staining. Representative results of flow cytometry for Mewo-Luc cells without (A) or with (B) PKH26 staining shown as dot plots.

Almost all live Mewo-Luc cells just after PKH labeling were positive for PKH and detectable by FCM (positive: 98.8 ± 0.5%) (Figure 2).

### 3.2 Validation of serial dilution for Mewo-Luc cells

**Figure 3:**
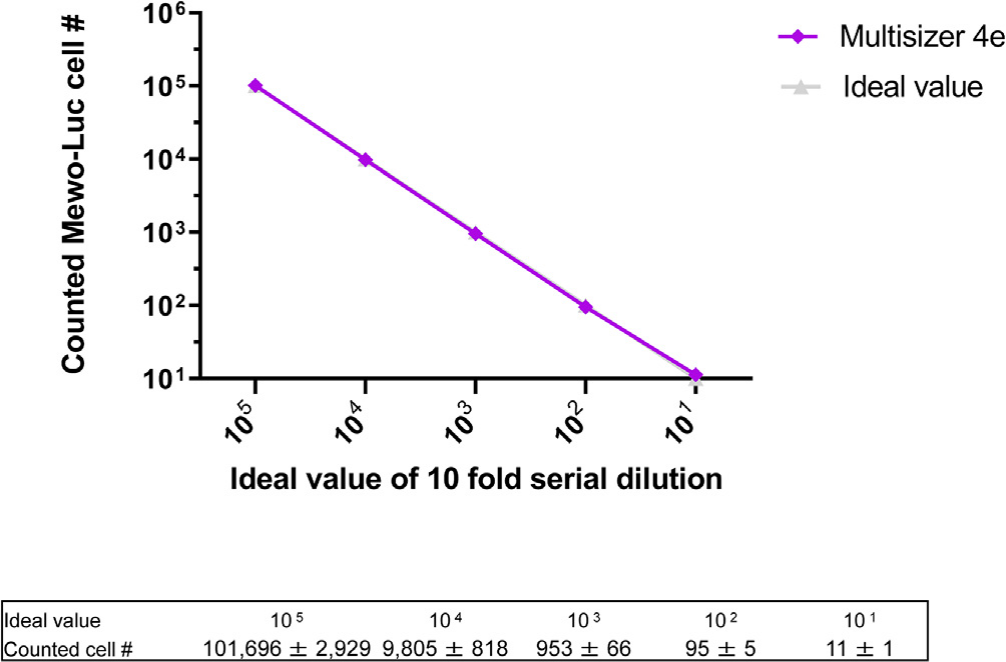
Validation of the accuracy of 10-fold serial dilution using a Coulter counter. Plots of ideal value of 10-fold serial dilution (X-axes) and counted cell number (Y-axes) (upper). Table of actual values (lower).

Average of Mewo-Luc cell diameter was calculated as 14.5 μm. Coulter counter counted original sample (counted as 2,000,000 cells/mL with hemocytometer) as 1,983,283 ± 20,852 cells/mL. Ten-fold serially diluted samples were counted and calculated as follows, 100,000 cells: 101,696 ± 2,929 cells; 10,000 cells: 9,805 ± 818 cells; 1000 cells: 953 ± 66 cells; 100 cells: 95 ± 5 cells; 10 cells: 11 ± 1 cells, respectively (Figure 3). These results indicate that 10-fold serial dilution technique were considered to be reliable which provides almost equivalent cell counts as ideal values with small deviations.

### 3.3 Quantification of positive cells in cell suspensions

**Figure 4:**
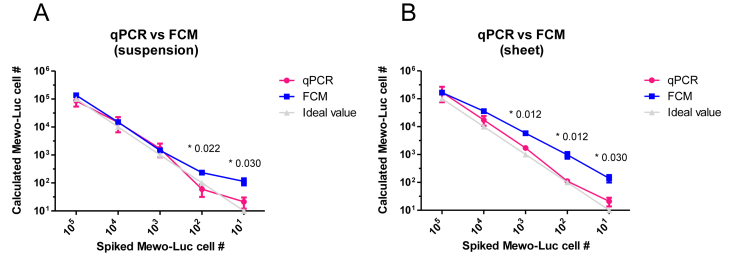
Comparison of accuracy of 2 methods. Comparisons of spiked cell number (X-axes) and calculated cell number (Y-axes) using qPCR and FCM in (A) cell suspensions and (B) cell sheets.

We spiked various numbers of Mewo-Luc cells (100,000 cells, 10,000 cells, 1,000 cells, 100 cells, 10 cells or 1 cell, respectively) in 500,000 of hMSC suspensions as described in Methods (*Preparation of hMSC suspensions and Mewo-Luc spiking*) (Figure 1A). Total cell counts of each sample and the percentages of spiked cells determined by qPCR and FCM are shown in Table 1A. Positive cell numbers included in spiked samples are calculated and plotted in Figure 4A. There was no significant difference in calculated positive cell numbers between both methods for the samples spiked with 100,000 cells, 10,000 cells and 1,000 cells, respectively. On the other hand, samples spiked with 100 and 10 positive cells indicated significantly higher cell numbers in calculations by FCM, respectively (qPCR vs FCM: 100 cells: 59 ± 25 vs 232 ± 35 cells, p = 0.022/10 cells: 21 ± 7 vs 114 ± 27 cells, p = 0.030). Neither test could detect cells in the experiments spiked with 1 cell.

**Table 1:** Collected cell number and percentage of each format. Average of total collected cell counts and percentages of spiked cells for each format of stem cell product; (A) cell suspensions, (B) cell sheets. qPCR, quantitative polymerase chain reaction; FCM, flow cytometry.

(A)					

Total collected cell count					

spiked #	100,000	10,000	1,000	100	10
	668,000 ± 52,566	524,000 ± 64,162	492,000 ± 28,199	496,000 ± 31,061	576,000 ± 87,159
% of spiked cell					
qPCR, %	12.41 ± 3.87	3.1 ± 1.56	0.36 ± 0.16	0.01 ± 0.01	0.004 ± 0.002
FCM, %	21.22 ± 3.45	3.00 ± 0.51	0.30 ± 0.06	0.05 ± 0.01	0.027 ± 0.003
(B)					

Total collected cell count					

spiked #	100,000	10,000	1,000	100	10
	256,000 ± 20,317	176, 000 ± 16,395	190,000 ± 15,232	190,000 ± 19,390	158,000 ± 17,297
% of spiked cell					
qPCR, %	59.35 ± 26.2	8.92 ± 2.33	0.9 ± 0.11	0.06 ± 0.01	0.01 ± 0.004
FCM, %	64.48 ± 5.73	20.0 ± 2.49	3.18 ± 0.5	0.51 ± 0.11	0.10 ± 0.03

### 3.4 Quantification of positive cells in cell sheets

We performed similar spiking of Mewo-Luc cells onto hMSC sheets as described in Methods (*Preparation of hMSC sheets and Mewo-Luc spiking*) (Figure 1B). Total cell counts of each sample and the percentages of spiked cells determined by qPCR and FCM are shown in Table 1B. Positive cell numbers included in spiked samples are calculated and plotted in Figure 4B. There was no significant difference in calculated positive cell numbers using qPCR and FCM in the samples spiked with 100,000 cells and 10,000 cells. Samples spiked with 1,000, 100 and 10 positive cells indicated significantly higher cell numbers in calculations by FCM, respectively (qPCR vs FCM: 1,000 cells: 1723 ± 258 vs 5810 ± 878 cells, p = 0.012/100 cells: 110 ± 18 vs 973 ± 232 cells, p = 0.012/10 cells: 20 ± 6 vs 141 ± 36 cells, p = 0.030). Neither test could detect cells in the experiments spiked with 1 cell.

## 4 Discussion

In the present study, we evaluated differences in the accuracy of positive cell counting methods in experimental stem cell products spiked with tumorigenic cells recapitulating cell sheet transplantation or injection of cell suspension. FCM significantly overestimated positive cell numbers compared to those from qPCR analyses in both formats of stem cell product when spiked with fewer cells. qPCR seemed to estimate more accurate positive cell number close to the ideal value.

Serial dilution is a commonly and historically used technique in the life science fields such as in cell biology, microbiology, and virology. To estimate accurate cell or microbial concentration by serial dilution, several estimation methods have been reported [13, 14, 20]. Previous reports evaluating tumorigenicity [10, 11, 21, 22] employed a theoretically calculated cell number by serial dilution without precise counting of cell numbers. To our knowledge, there is no report experimentally evaluating the accuracy of serial dilution technique for single cell suspensions so far. Although the results might be affected by several factors such as operator variability, pipetting technique, temperature, cell viscosity and so on, the results of cell counting for serially diluted cell suspensions using a Coulter counter in the present study might have provided a fundamental information on the accuracy of serial dilution of the cell suspensions.

The present study indicates the importance of the accuracy of each method for the quantification of target cells, especially in cases of small cell numbers, in designing a tumorigenicity study model. Droplet digital PCR technology has been recently introduced for more accurate evaluation of cell count and this approach may provide more precise and reproducible results [23, 24].

Kuroda and colleagues reported [21] a high sensitivity assay for the detection of residual undifferentiated human induced pluripotent stem cells (hiPSCs) among retinal pigment epithelial cells derived from hiPSCs. The authors evaluated quantitativity of undifferentiated cells using undifferentiated cell markers such as TRA-1-60 and Lin28. In the present study, we used malignant melanoma cells as a positive control to recapitulate malignant transformation. It is still unclear whether our results can be applied in the detection of residual undifferentiated hiPSCs as well using markers such as TRA-1-60 and Lin28, and should be evaluated as our next work.

In conclusion, we have evaluated the accuracy of commonly used methods on the quantification of spiked tumorigenic cells in 2 classes of stem cell products; cell suspension and cell sheet. Differences in accuracy between quantification methods should be considered in designing a tumorigenicity study model. The concept might be broadly applied to pre-clinical safety tests of stem cell therapies for various target organs.

## Declarations

### Author contribution statement

Hiroaki Osada, Hidetoshi Masumoto: Conceived and designed the experiments; Performed the experiments; Analyzed and interpreted the data; Wrote the paper.

Masahide Kawatou: Performed the experiments; Analyzed and interpreted the data.

Masafumi Takeda, Jun-ichiro Jo: Performed the experiments.

Takashi Murakami: Contributed reagents, materials, analysis tools or data.

Yasuhiko Tabata, Kenji Minatoya, Jun K. Yamashita: Conceived and designed the experiments.

### Funding statement

This work was supported by grants from 10.13039/100009619Japan Agency for Medical Research and Development (AMED) under Grant Number JP18he0702246 (to Y.T., K.M., J.K.Y. and H.M.).

### Competing interest statement

The authors declare no conflict of interest.

### Additional information

No additional information is available for this paper.
